# Role of the Menstrual Cycle on Performance and Injury Risk: A Survey of Female Professional Rugby Players in the United Kingdom

**DOI:** 10.3390/ijerph21020150

**Published:** 2024-01-29

**Authors:** Eloise Hayward, Liz Akam, David Hunter, Sarabjit Mastana

**Affiliations:** School of Sport, Exercise and Health Sciences, Loughborough University, Loughborough LE11 3TU, UK; eloisehayward@icloud.com (E.H.); e.c.akam@lboro.ac.uk (L.A.); d.j.hunter@lboro.ac.uk (D.H.)

**Keywords:** menstrual cycle, rugby players, injury risk, performance, education, thematic analysis

## Abstract

Background: Female athletic performance and injury risk is impacted by variations in the menstrual cycle (MC), but the understanding of the impacts and mechanisms influenced by the menstrual cycle on exercise performance are not fully delineated. Aims and Objectives: Evaluate associations between the menstrual cycle, perceived performance, and injury risk of elite female rugby players using an online survey. Methods: An anonymous online questionnaire was completed by 150 elite female rugby players from two English rugby leagues, the Betfred Women’s Super League (BWSL) and the Allianz Premier 15s (AP15s). The collected data were analysed thematically. Results: The Chi-square test was used to assess associations between age groups and contraception usage, weight change, and training and playing performance; none of the associations were statistically significant (all *p* values > 0.05). Thematic analysis of 11,660 words of data revealed four themes: (a) MC impact on training and competition, (b) education and period management plans, (c) openness of conversations and comfort taking time off, and (d) injury risk. The impacted performance areas were physical (83.7%), psychological (85.7%), and nutritional (80.3%); players experienced decreased appetite, nausea, fatigue, strength declines, heighted emotions, and worsened focus. In total, 87.8% of athletes perceived the MC to negatively impact performance, 85.7% of players desired to be educated further to prevent injuries, improve nutrition, and training adaptions, 51.7% of participants perceived risk of injury to be higher during MC, and 86.4% of participants did not feel comfortable taking time off due to the MC, worrying that selection would be affected and about opinions from others. Conclusion: A clear negative impact on perceived performance and injury risk was reported by survey participants. The interaction of physical, psychological, and nutritional factors, and a lack of awareness and education emphasise the need for further comprehensive studies and interventions, with measures such as MC monitoring and profiling, education, and training adaptions to develop openness, knowledge, and understanding.

## 1. Introduction

Female participation in sport, as recreation and a profession, has increased globally over the last two decades [1]. Women’s sport has produced the greatest increase in television audiences worldwide, with women’s rugby being one of the most popular [2]. Research into the risks of menstrual dysfunction and relative energy deficiency (RED-s) is growing, however performance recommendations are largely based on research using male participants [3]. A large proportion of female athletes are impacted by a variation of the menstrual cycle (MC), but studies on perceived impact on performance and injury among different sporting populations are relatively limited; expanding research will support the understanding of the impacts and mechanisms influenced by the MC on exercise performance and will guide the adoption of training, recovery, and athlete monitoring regimes [4].

The MC starts with menarche, normally between the ages of 10 to 16, and continues regularly until the menopause, at an average age of 51, if not interfered with by pregnancy, the use of hormonal contraceptives (HC), menstrual or ovulatory dysfunction [5]. The MC is characterised by fluctuations in reproductive hormones: estrogen, progesterone, follicle stimulating hormone (FSH), and luteinizing hormone (LH) [6]. Premenstrual and menstrual symptoms can affect training, competing, and day to day living of athletes. Heavy periods known as menorrhagia can cause iron deficiency anaemia which manifests as constant fatigue, weakness, dizziness, and shortness of breath [7]; even mild iron deficiency anaemia impairs maximal performance [8].

MC related symptoms are common, particularly dysmenorrhea and premenstrual symptoms, which influence perceived performance of aerobic fitness, speed, muscle strength, power, mental sharpness, balance, and sleep quality [9,10,11,12,13,14]. Many athletes believe their performance is influenced by menstrual disturbances [9,10,11,12,13,14], particularly in the early follicular and late luteal phases, which coincide with the occurrence of menstrual symptoms [11,15]. Fatigue or lethargy [10,16] were frequently mentioned as causes for this perceived performance decline. Hormone concentrations impact rapid force production, necessary for explosive movements, affecting muscle strength and power [4,6]. It was suggested that muscle strength and maximal and submaximal aerobic performance, are reduced during the early follicular phase (EFP) due to low levels of estrogen and progesterone compared with other MC phases [17]. Changes in body composition throughout the MC may also contribute to altered performance. Increased body mass is linked to impaired aerobic and anaerobic endurance capacity [18,19,20,21,22,23]. Female athletes experience injuries at a 3–8× higher rate than male athletes [24]. The late follicular phase, where estrogen is high, is when international football players are almost twice as likely to sustain muscle and tendon injuries [25].

Menstruation is suggested to cause a magnitude of psychological effects, including over 100 psychosomatic symptoms such as anxiety and depression [26]. Over 70% of women complained of premenstrual irritability, decreased morale, depression, fatigue, abdominal bloating, or backache [27]. Fluctuations in the hormones estrogen, testosterone, and aldosterone can impair performance in spatial ability and cognition tasks [28]. Progesterone has sedative properties [27], which can prevent athletes from performing to their full potential. Bale et al. documented that 59% of athletes experienced a decrease in performance during the late luteal phase (LLP) due to premenstrual symptoms [29]. Also, menstrual stigma is suggested to be a global educational and socioeconomic problem with 56% of 14-year-old girls feeling embarrassed by their period [30]. 

MC phase monitoring is being used more frequently in professional sports. Fitr Woman, a smartphone application, has been used to track players’ MCs by elite sporting organisations such as Chelsea Football Club and the United States Women’s National Soccer Team (USWNT) who linked MC monitoring and individualised support to World Cup victory [31]. Athletes use the app to track their MC and record occurrences of menstruation and various symptoms. The information recorded can be accessed by staff to determine objectively whether an athlete’s performance or readiness changed throughout the MC or deviated from their typical cycle. Individual solutions were created for variations in sleep, recovery, and performance based on the application’s recommendations [32,33,34]. Individual solutions are essential, as Olympic medal-winning performances have occurred during the MC, while other athletes have missed out on achievements [32,33]. 

Research Aims: As comprehensive studies on the understanding of the role and effects of MC among rugby players are lacking, this questionnaire-based cross-sectional study was designed with the aim of investigating the association between the MC and perceived performance and injuries in elite female rugby players. It focuses on multiple factors that influence performance, such as nutrition, psychology, and physical parameters, and examines current period education and further education requirements of players and staff. This would eventually pave the way towards finding a relationship between the MC and sports performance and injuries, which could be applied in practice to reduce the number of injuries and decreases in performance.

Research Objectives:

1. Investigate the perceived effects the MC has on athletic performance and injury in professional rugby players in the UK.
2. Identify themes and subthemes which athletes consider important in their athletic performance and injury.
3. Assess the level of MC education, concerns, and support processes available.

## 2. Methods

### 2.1. Study Design

Professional female rugby players (aged ≥ 18 years) playing in the Betfred Women’s Super League (BWSL) (6 teams of 30 members = 180) or Allianz Premier 15s (AP15s) (10 teams of 30 members = 300) during the 2022/23 season completed an anonymous online questionnaire. The total available pool of participants was approximately 480 participants. To ensure that only the desired participants completed the questionnaire, an online link of the anonymous questionnaire was sent directly to WhatsApp groups of AP15s and BWSL teams. The questionnaire comprised 25 questions and 3 main sections: (1) Period data; (2) MC and performance experience; and (3) Comfort in discussing MC and education. Pilot testing for both content and face validity was performed [35]. The questionnaire was created in Online Surveys (Bristol, UK) and was open for 8 weeks beginning January 2023. Loughborough University Ethics Review Sub-Committee approved the study (Project ID:13281). All participants provided informed consent prior to completing the questionnaire. Individual athletes were not identifiable at any stage of the research. The questionnaire used in this study is available as a Supplementary Document. 

### 2.2. Data Analysis

Questionnaire results were collated into a Microsoft Excel spreadsheet and exported to SPSS (version 28.0). Only completed and consented questionnaires (*n* = 147) were included in the final analysis. Data from three participants were excluded as full consent was not provided. Chi-squared (***x***^2^) tests were carried out to examine the relationship between categorical variables such as age, contraception users, and weight change. Data were represented as mean, frequencies, and percentages, and the threshold for statistical significance was set at *p* ≤ 0.05.

Responses to open-ended questions were analysed according to thematic analysis using the Braun and Clarke’s 6-step model as a means of identifying, analysing, and reporting common patterns within the data [36,37]. Identifying the main themes through familiarisation, open coding, and grouping raw data was performed by hand by the main investigator (EH), working through hard/Excel copies of the comments [36,37]. 

## 3. Results

Data from 147 participants were used for further analysis. The completion rate for the survey was 31%. Overall, approximately 11,660 words of text were utilised in thematic analysis. The analysis uncovered four main themes as below and 28 sub-themes (Table 1):

(a) MC impact on Training and Competition;
(b) Education and Period Management Plans;
(c) Openness of Conversations and Comfort Taking Time Off;
(d) Injury Risk.

Descriptive menstrual cycle data, hormonal contraceptive use, and participant characteristics are shown in Table 2.

**Table 1:** Themes and Subthemes identified via thematic analysis [36,37].

Overarching Themes	Sub Themes
Impact on Training and Competition—Physical, Psychological, Nutritional	Fatigue Strength Speed/Agility Concentration/Focus Pain from Symptoms Flooding Worries—White Shorts Anxiety/Stress/Motivation/Emotional Endurance Nausea Change in Appetite Cravings Increased Thirst
Education/Consideration	Nutrition Injury Prevention Gym/Load Psychological Fatigue Medications and Symptom Management Coach Awareness Lifestyle Changes
Openness of Conversations/Taking Time Off	Staff Gender Staff Role Affect Selection Opinions/Misunderstood by Players & Staff Internal Opinion/Conflict Financial
Injury Risk	Weaker/Less Athletic Injury History (themself or teammate) Concentration/Energy/Confidence Sore Muscles/Lower Pain Threshold

Athletes were asked to rate 13 different symptoms on a scale of 1–5, with 1 being no effect on performance and 5 being dramatic effect. Based on averages, fatigue (3.28), mood swings (3.27), and abdominal cramps (3.26) were ranked as the highest effect symptoms (Table 3). 

The Chi-square test to assess associations between age groups, contraception usage and weight change, and training and playing performance revealed no significant associations (*p* > 0.05). 

### 3.1. MC and Performance Impact in Physical, Psychological, and Nutritional Domains and Associated Qualitative Comments

A total of 129 athletes (87.8%) reported impacted performance, categorised into physical, psychological, and nutritional impacts. Key performance concerns were fatigue (n= 53, 22.9%) and strength (weaker) (n = 48, 18.6%) (Table 4). Key quotes included ***“Paranoia and discomfort makes me lose focus. Stomach cramps and pain as well as higher fatigue make training more difficult, not as explosive as normal”*** and ***“I feel less motivated, more tired and it’s hard to manage your period on an intense training schedule*”**. Further qualitative comments/quotes are included in Supplementary Document S1. 

Psychologically, 126 athletes (85.7%) were affected. Symptoms manifested as anxiety, stress, and feeling more emotional (n = 44, 36.7%), followed by worries over bleeding through match kit (n = 44, 25.3%) and concentration (n = 41, 23.6%). One participant said ***“White shorts can have a big impact as there is always a fear of leaking through clothes, but this fear is maximised when wearing white shorts. Also, the feeling of being unhygienic on match day having to go to the toilet several times before kick-off, it can be stressful to cope with on top of match day”.***

Physically, 123 athletes (83.7%) were influenced. Strength (n = 61, 33.5%) and speed (n = 58, 31.9%) were the common areas worsened. One participant quoted ***“Muscles fatigue easier making gym and training on the pitch harder. Feels like reactions are slower physically”.***

Nutritionally, 118 athletes (80.3%) were impacted. An increase in consumption of sugary and salty foods (n = 48, 35.6%) and nausea (n = 25, 18.5%) were the main contributors (Table 4). Participants quoted ***“Increased appetite for foods that aren’t necessarily the best for fueling performance sugary and salty foods which satisfy cravings”.***

Overall performance was worse for 103 athletes (70.1%), while 41 (27.9%) stayed the same. Reasons for worsened performance were feeling less athletic (n = 37), fatigue (n = 30), and a lack of concentration (n = 25). Weight fluctuations occurred during the MC with 116 (78.9%) athletes increasing, 6 (4.08%) decreasing, and 25 (17.0%) staying the same. Main qualitative quotes included ***“Coordination, power and endurance are worse and more anxious/fearful of contact situations”; “I feel the pain and discomfort I experience on my period impacts my performance as I feel weaker, slower and paranoid of leaking through my shorts.”***

An increased risk of injury during the MC was felt by 76 athletes (51.7%). Previously, 56 athletes (38.1%) had been injured during their period or the days leading up to/after it. Injuries ranged from knees (39.5%) and ankles (28.9%) to fractures/broken bones (10.5%). More specifically, ACL tears (19.6%), Anterior Talo-Fibular Ligament (AFTL) tears (19.6%) or meniscus tears (10.7%) were reported. Reasons for increased susceptibility to injury stemmed from feeling less athletic and weaker (32.1%), a lack of concentration, energy, and confidence (28.3%), and previous injury to themselves or a teammate (28.3%) (Table 4). One player quoted “***Bucket handle medial meniscal tear. First time was 9 months [recovery], second time was 4–5 months [recovery]. I believe the first time was the stage of my menstrual cycle where I was near ovulation and the elasticity in my joints and ligaments was increased”.***

### 3.2. Education

Period management education around performance would benefit 126 athletes (85.7%) and 113 (76.7%) would value period consideration in training loads (Table 5). Out of 147 athletes, 66 had “Some” (44.9%), 44 had “Not a lot” (29.9%), and only 26 (17.7%) “Had enough” education on periods. This education came from school for 105 athletes (41.8%) and self-research for 80 (31.9%) (Table 5). Qualitative comments included ***“Greater awareness for coaches so they understand you’re not having a bad performance through lack of effort. Lifestyle changes or load management suggestions would be good, S&C adaptations in programs would be helpful”*** and ***“Ways to manage nutrition, training load* etc. *And just coaches being aware that people need time to be able to manage their period* e.g., *toilet breaks and not being rushed”.***

### 3.3. Openness of Conversation 

A total of 81 athletes (55.1%) were comfortable speaking to a member of staff regarding the MC effects on performance. Although the gender of staff spoken to differs, 60.6% speak to only female staff whilst 33.3% speak to either gender. The job roles of staff had an impact on conversation; physios (41.6%), specifically female physios (26.5%), were more likely to be spoken to. A total of 127 athletes (86.4%) were not comfortable taking time off due to the MC effects. Reasons for this were opinions from other athletes and staff (45.0%) and affecting selection into the team (23.3%) (Supplementary Table S1).

## 4. Discussion

This study assessed the impact of MC on performance, injury risk, and other factors in an elite rugby sample from England. As this is a small study, caution is warranted in interpretations, but some actionable outcomes should be generalisable to rugby players and different sporting communities. 

### 4.1. Performance 

An overwhelming 87.8% of athletes believed the MC to affect performance. Previous research found similar results; 77% of elite athletes had negative side effects during the MC [38,39]. 

A negative impact on multiple performance areas was perceived: psychological, physical, and nutritional. Fatigue and abdominal pain were the most common complaints; whilst athletes showed a reluctancy to take time off, it was determined that pain affects performance [39]. Further research is needed to determine if these results are a chance occurrence or evidence that elite athletes are more accustomed to pain [11,39].

Rugby players lack psychological support; 85.7% of performances were negatively impacted by worsened confidence, focus, and heightened emotions [40]. Employing strategies suggested in this article could avoid impacts: lack of knowledge and self-management skills addressed by basic education; open communication, acceptance, and normalisation of menstrual-related issues among teammates and staff to rectify irritation, altered mood, and embarrassment. Rugby players wear a pre-determined, standardised kit such as white shorts, but 25.3% of athletes reported paranoia of flooding. A study on gymnasts reported similar results of intrusive thoughts, heightened anxiety, and reduced attentional focus [41]. Females tend to change clothing to manage and conceal symptoms, particularly excessive blood loss. However, this is not always possible for athletes, thus feeding worries and distractions [42].

Physical demands have increased as rugby has become faster and more intense [43]. Speed and strength were most affected by the MC. Research suggests tailored training to prevent impacts. Delayed onset muscle soreness and strength loss are highest in the early follicular phase (EFP) and lowest in the mid luteal phase (MLP). It is recommended that lower training loads or longer recovery periods could be considered in the EFP and strength conditioning loads could be enhanced in the MLP [43,44,45,46]. Chelsea became the first club in 2020 to modify training according to menstruation; since then they have won the last two league titles [19]. Athletes associate a lack of readiness to compete with fatigue, sore muscles, and a lack of concentration. Warm-ups are a key component of performance but are negatively impacted by menstrual symptoms [46]. Intervention could include longer focused warm-ups for athletes at detrimental cyclical stages or speed and reaction sessions [43]. It is advisable and beneficial to track the MC, knowing when to tailor training, preparing to manage symptoms, and predicting peak player fitness.

Fatigue is mentioned in every category as a negative impact. Previous studies linked increased perception of tension-anxiety and fatigue in the LLP with lower levels of serotonin, suggesting low estrogen concentrations are responsible for this [19,46,47,48,49]. Considering that estrogen remains relatively low in the EFP, then serotonin production will remain low, and fatigue will be increased. 

Craving sugary and salty foods (35.6%) was the primary nutritional effect. Changes in cravings, appetite, and energy intake during the MC aligns with fluctuating serotonin concentrations. Low serotonin activity is associated with food cravings and overconsumption [23]. Weight gain may also occur as aldosterone elevation causes water retention alongside increased consumption of highly salted foods [50]. A total of 25 athletes reported nausea and 24 athletes reported decreased appetites altering their nutritional regime, a crucial aspect of optimal performance; smaller meals with higher nutritional value could be incorporated [51]. Despite women having cyclical variations in dietary requirements, research into athlete nutrition is male dominated; additional research is needed to understand female nutritional needs [52,53]. Increased body mass is associated with impaired aerobic endurance performance and anaerobic performance [53,54]. It is not coincidental that 78.9% of athletes’ weight increased over the MC and 70.1% of athletes stated their performance was worse. 

### 4.2. Education 

Period education around performance would benefit 85.7% of athletes. Alarmingly, 44.9% of athletes only had “some” education and 29.9% had “not a lot”. Sources of education were school and self-research; sport only accounted for 19.9% highlighting a need to educate athletes. A total of 55.1% of athletes felt comfortable speaking to a member of staff about MC impacts. However, 60.6% would only speak to a female member of staff, specifically a female physio (26.5%). 

Improving athlete and coach knowledge will significantly reduce the discomfort of MC conversations [55]. This is important in shifting mentality, improving treatment, understanding, athlete wellbeing, and performance [8]. Coaches have shown a willingness to be educated specifically in training management and physical performance during the MC [56,57]. Educational materials should provide strategies to discuss menstrual health issues with athletes in a comfortable and sympathetic manner.

Careful consideration of match day schedule, including overnight stays to avoid travel, increased toilet breaks, more time in the changing room before kick-off, and the provision of painkillers and sanitary products could minimise negative experiences, allowing athletes to feel physically and psychologically prepared to play. Financially there may be a barrier as female teams have less funding than their male counterparts. Research into the MC against performance may lead to increased investment from clubs into MC management and education [58]. 

A total of 86.4% of athletes were uncomfortable taking time off due to their period. It was concluded that although athletes accept MC symptoms, they do not consider them a reason to refrain from training [11]. In contrast, women in the general population limit everyday activity during menstruation [59]. Athletes are expected to be tough due to the “suck it up” and pain principles in elite sport [59,60]. Contact sport athletes induce pain from impact and exertion, perhaps expecting pain, hence MC symptoms become tolerable. This mindset, along with pressure to perform, may lead to athletes adopting an “accept and adapt” attitude [11]. 

### 4.3. Injury Risk 

An increased risk of injury during the MC was felt by 51.7%; the main reasons were: feeling less athletic and a lack of concentration. Ovarian hormones influence the speed of decision-making and cerebral function, increasing the risk of accidents. A total of 56 participants reported having been injured during an active MC with the most common sites being the knees and ankles. Previous studies suggest the higher ligament injury rate in the late follicular phase (LFP) is down to reduced ligament and tendon stiffness which compromises joint stability [60,61,62]. Estrogen concentrations are highest in the LFP which have been shown to be negatively associated with tendon stiffness [63,64,65]. Studies have discovered that female connective tissue expresses hormone receptors for estrogen and progesterone; for example, the ACL possessing 17-b estradiol receptors [66]. Relaxin, a hormone which relaxes soft tissues, appears to rise during day 12, peak at day 14, and again halfway through the luteal phase [66]. This could contribute to laxity where athletes have stated feeling “unstable”.

This study has certain limitations: It is not representative of World Rugby as the questionnaire was only available in English and circulated in two English domestic leagues with responses only collated from players in the United Kingdom. The population was senior professional women rugby players; therefore, results may not be transferrable to rugby teams of different performance levels or ages, nor to different sports. As the study consisted of one point of data collection, results may not have been representative of a typical week whilst menstruating. Self-report studies suffer recall bias as each athlete may have completed the questionnaire at a different phase of the MC and this may have influenced their perceptions. Ethnic and cultural heritage questions were not included on the questionnaire which is a limitation to the investigation of these additional factors. These may prove to be significant factors/influences for further research [67]. Also, information on the use of hormonal contraceptives and their effect on MC and performance was not comprehensively explored in this study. The severity of pain is limited by memory and once the experience ends, the reliability of pain recall reduces [68]. Due to the nature of the questionnaire design, only a generalised level of athlete perceptions was evaluated rather than a detailed examination. Individual interviews may have enhanced the depth of collected information. 

There are several strengths to this study. Firstly, quantitative and qualitative measures of performance throughout the MC were examined with the use of a questionnaire, an inexpensive method that has advantages of precision and accuracy, as well as being easy to use. 

## 5. Conclusions

This study marks the exploration into performance perceptions of elite female rugby players during the MC. Athletes experiences varied; however, almost all reported effects in physical, psychological, and nutritional performance. The effects should be addressed through altered training schedules, cycle monitoring, and education. However, caution is warranted as these suggestions are based on the results of a UK sample with a limited number of participants. Education will allow positive conversations between staff and athletes, removing anxiety and normalising menstrual issues, essential to eliminate MC stigma. There may be a degree of difficulty implementing the management strategies identified due to limited research, resources, and finances at women’s clubs. Players’ cycles differ in time scale, impact, and symptomology thus an individualised approach by specialists may be the gold standard. 

## Figures and Tables

**Table 2:** Participant Characteristics, Menstrual Cycle Characteristics, Tracking Data, and Hormonal Contraceptive Data.

Participant Characteristics	Participant (Nos.)/Parameter	Percentage
Mean Age	23.1 ± 3.5	
Participants aged between 18–24	101	68.7%
Participants aged between 25–31	38	25.9%
Participants aged between 32–38	8	5.44%
**Menstrual Cycle Characteristics**		
Mean frequency of menses (days)	29.9	
Mean duration of menses (days)	4.97	
Participants who have gone without regular menstruation	54	36.7%
**Reasons for not menstruating**		
Exercising too much	24	32.9%
Stress	15	20.6%
Inadequate nutrition	11	15.1%
**Tracking data**		
Participants tracking their menstrual cycle	107	72.8%
Apps	66	82.5%
Watches	10	12.5%
Calendar	4	5%
**Hormonal contraceptive data**		
Hormonal contraceptives	33	22.4%
Type of contraceptive used	Combined pill; n = 13 Coil; n = 10 Implant; n = 6 Injection; n = 1 Patch; n = 1 Progesterone only pill; n = 1 Oestrogel; n = 1	39.4% 30.3% 18.2% 3.0% 3.0% 3.0% 3.0%

Not all participants stated how they tracked their MC or reason for not menstruating.

**Table 3:** Symptoms ranked in order of most effect on training and playing performance to least based on averages. Effect size rating was 1—no effect, 2—some effect, 3—noticeable effect, 4—significant effect, and 5—dramatic effect.

Symptom	Average
**Fatigue**	3.28
**Mood swings**	3.27
**Abdominal cramps/pain**	3.26
**Decreased motivation**	3.23
**Feeling irritable**	3.22
**Lower back pain**	3.02
**Tender breasts**	2.89
**Bloating**	2.89
**GI issues (constipation, diarrhoea)**	2.70
**Joint pain**	2.62
**Headaches**	2.29
**Trouble sleeping**	2.20
**Acne**	2.05

**Table 4:** MC and its effects in different domains.

Q: Do You Consider Your Training/Playing Performance to Be Affected During Your Period?	Participants	Percentage
Yes	129	87.8%
No	18	12.2%
**Performance effect themes identified**		
Fatigue	53	22.9%
Weaker	43	18.6%
Slower	37	16.0%
Concentration/Focus	26	11.3%
Mental (motivation, stress)	38	16.5%
Pain from symptoms	34	14.7%
Total	231	
**Q: Does Your Period Psychologically Affect Performance?**		
Yes	126	85.7%
No	21	14.3%
**Psychological effect themes identified**		
Paranoia about kit	44	25.3%
Emotional (anxiety, stress)	64	36.7%
Concentration	41	23.6%
Fatigue	25	14.4%
Total	174	
**Q: Does Your Period Physically Affect Your Performance?**		
Yes	123	83.7%
No	24	16.3%
**Physical effects identified**		
Endurance	31	17.0%
Speed	58	31.9%
Strength	61	33.5%
Fatigue	32	17.6%
Total	182	
**Q: Does Your Period Nutritionally Affect Your Performance?**		
Yes	118	80.3%
No	29	19.7%
**Nutrition effects identified**		
Nausea	25	18.5%
Increased appetite	22	16.3%
Decreased appetite	24	17.8%
Crave sugary and salty foods	48	35.6%
Increased thirst	16	11.9%
Total	135	
**Q: Do You Consider Your Performance to be Better, Worse, or Same on Your Period?**		
Better	3	2.04%
Same	41	27.9%
Worse	103	70.1%
**Q: Does Your Weight Change During Your Menstrual Cycle?**		
Yes—Increases	116	78.9%
Yes—Decreases	6	4.1%
No—Stays the same	25	17.0%
**Q: Do You Feel More at Risk of Injury on Your Period?**		
Yes	76	51.7%
No	71	48.3%
**Reasons for Feeling More at Risk of Injury**		
Weaker/Less athletic	17	32.1%
Injury (to themself or someone they know)	15	28.3%
Concentration/Energy/Confidence	15	28.3%
Sore Muscles/Lower Pain Threshold	6	11.3%
**Q: Have You Ever Been Injured Whilst on Your Period or About to Start?**		
Yes	56	38.1%
No	91	61.9%
**Main Injuries Reported**		
Knee	15	39.5%
Break/Fracture	4	10.5%
Ankle	11	28.9%
Hamstring	1	2.6%
Calf	1	2.6%
Nerves	1	2.6%
Concussion	3	7.9%
Back	1	2.6%
Dislocation	1	2.6%

Note: The numbers on themes and effects are variable as participants were allowed to choose multiple effects.

**Table 5:** Period Management Education, Training/Playing Loads and Generic/Individual Plans.

Q: Do You Think You’d Benefit from Period Management Education around Performance?	Participants (Numbers)	Percentage
Yes	126	85.7%
No	21	14.3%
**Q: Would You Like Your Period to Be Considered in Training/Playing Loads?**		
Yes	113	76.7%
No	34	23.1%
**Q: Would You Like a Generic/Individual Plan from Staff in Place to Manage Your Period?**		
Yes	90	61.2%
No	57	38.8%
**Education Categories**		
Nutrition—Food Choices Pre and Post Training and Anti-Inflammatory Foods	48	16.2%
Injury Prevention/Prehab—Pelvic Floor Work, Exercises for Susceptible Injuries	55	18.6%
Gym And Training Load—Adaptations to Programmes for Weights, Sets, Reps, Intensity, and Sessions Completed	52	17.6%
Psychological and Concentration	26	8.8%
Fatigue	50	16.9%
Medications	22	7.4%
Coach Awareness and Understanding	21	7.1%
Lifestyle Changes—Daily Routine Adaptions, Sleep Quantity and Quality, Mindfulness	22	7.4%
Total	296	
**Training/Playing Load Categories**		
Understanding Staff/Open Conversations	21	24.7%
Reduced Training Loads/Intensity	48	56.5%
Altered Gym and Training Targets	16	18.8%
Total	85	
**Generic/Individual Plan Categories**		
Gym and Training Load	34	50.7%
Nutrition	12	17.9%
Understanding Staff	9	13.4%
Symptom Management	12	17.9%
Total	67	
**Q: How Much Education Have You Had on Periods?**		
None	5	3.40%
Not A Lot	44	29.9%
Some	66	44.9%
Enough	26	17.7%
A Lot	6	4.1%
Total	147	
**Where Was It From?**		
School	105	41.8%
Sport	50	19.9%
Work	11	4.40%
Self-Research	80	31.9%
Other	5	2.0%
Total	251	

## Data Availability

Data analysed in this study is available from the 1st Author on request.

## Supplementary Materials

The following supporting information can be downloaded at: https://www.mdpi.com/article/10.3390/ijerph21020150/s1, Document S1: “Selected Qualitative Comments/Quotes in different domains/themes”; Table S1: ”Openness of Conversation, Gender, Role and Comfort Taking Time Off; Document S2: “Copy of the Online Questionnaire”.
