# Supplementary material for: Role of the Menstrual Cycle on Performance and Injury Risk: A Survey of Female Professional Rugby Players in the United Kingdom
Source: Int J Environ Res Public Health. 2024 Jan 29;21(2):150. doi: 10.3390/ijerph21020150 (PMC10888092; doi:10.3390/ijerph21020150)
Supplement: Supplementary file 1 [file ijerph-21-00150-s001.zip › ijerph-2771011-supplementary.pdf]

Supplementary Document 1

Selected Qualitative Comments/Quotes in different domains/themes.

Performance

- "No energy Running causes cramps Emotional state is all over the place".
- "Increased soreness and slower recovery between sessions, greatly increase fatigue".

Psychological

- "Awareness/ worry about changing tampon or bleeding through shorts/ tampon string being visible etc."
- "Light coloured shorts, less motivated, unpredictable moods, anxiety anticipating the effect the period could have on performance".
- "More emotional, often struggle with confidence".
- "Trouble understanding drills, sometimes it takes me longer to concentrate".

Physical

- "Particularly on first day(s), I feel slower and weaker and aware of not feeling good, which makes it hard to train at 'full' capacity".
- "Normal weights feel heavier"; and "Less powerful and my endurance levels feel they take a knock".

Nutritional

- "Because I feel bloated and nausea more often, I am sometimes not sure if I have eaten enough or not. This especially affects my pre-game or pre-training nutrition".
- "Have less of an appetite for balanced meals, crave the easy option".
- "How much I eat changes with each cycle. Some months I consume more and others less. I will usually drink more/feel thirsty".

Injury Risk

- "I ruptured my ACL a couple of days after I finished my period. Generally, the week after my period I have less energy and I feel like I might have under fuelled [due to symptoms]. Since surgery, I have also realised that my legs/knees feel weaker the days/week after menstruation".
- "In the gym, I feel less stable. For example, when completing a back squat, I'm more likely to rotate inwards at the knee. I feel slightly weaker".
- "I feel less stable, so I'm more vulnerable to sprains or tears in my joints. Specifically, my ankles or knees".
- "Hesitation in contact situations as I pre-emptively back off due to the abdominal pain already caused by the period, this hesitation could cause injury".

Education

- "Running load overall along with weight goals in the gym etc patterned or programmed around your cycle".

- “A gym and training programme that reflected my period. I.e., strength training at certain points and changes in intensity”.
- “I’d liked it to be treated the same as when you’re feeling more fatigued after a game or competition. Typically, you wouldn’t do an intense session if you’ve just played the day before so why would I do one if I’m feel really tired or weak”.
- “General information would be helpful for many players; however, I think the individualistic approach would be most beneficial for the player”.
- “Doesn’t have to be a huge plan. Maybe just an adaptation to a session or other exercises that might relieve symptoms.”

Supplementary Table S1: Openness of Conversation, Gender, Role, and Comfort Taking Time Off

**Do You Feel Comfortable Speaking to a Member of Staff Regarding Your Period and Cycle Effects?**	**Participants**	**Percentages**
Yes	81	55.1%
No	66	44.9%
**Gender of Staff Comfortable Speaking to**	**Participants**	**Percentages**
Male	4	6.10%
Female	40	60.6%
Either	22	33.3%
**Job Role of Staff Comfortable Speaking to**	**Participants**	**Percentages**
Physio	42	41.6%
Doctor	12	11.9%
Coach	14	13.9%
Manager	4	4.00%
Strength and Conditioning	12	11.9%
Nutritionist	3	3.00%
Any Role	14	13.9%

**Gender and Job Role of Staff Comfortable Speaking to**	**Participants**	**Percentages**
F Physio	44	26.5%
M Physio	21	12.7%
F Doctor	15	9.04%
M Doctor	11	6.63%
F Coach	16	9.64%
M Coach	3	1.81%
F Manager	8	4.82%
M Manager	0	0.00%
F Strength and Conditioning	15	9.04%
M Strength and Conditioning	12	7.23%
F Nutritionist	7	4.22%
M Nutritionist	1	0.60%
Any Role Any Gender	13	7.83%
**Do You Feel Comfortable Taking Time Off Due to Your Period?**	**Participants**	**Percentages**
Yes	20	13.6%
No	127	86.4%
**Categories of Comfort/Discomfort with Taking Time Off**	**Participants**	**Percentages**
Affect selection	42	23.3%
Opinions from others/misunderstood	81	45.0%

Opinions of self	41	27.8%
Financial	16	8.89%
Total	180	
**Quotes**	• *"Any female member of staff or male medical staff, I don't think I'd feel particularly comfortable discussing it with male coaching staff."*<br>• *"I will speak to anyone who feels comfortable having the conversation."*<br>• *"Struggle with selection, all male coaching staff are not understanding or sympathetic to the difficulties of training and work balance with a full-time job let alone with period/cycle issues to add in there".*<br>• *"Affect selection, feel like letting the team down, hate accepting the fact my period does and will affect my performance".*<br>• *"If it affects you that much it should be treated like any injury or illness, and you shouldn't train. Perceptions will only change if it's taken seriously".*<br>• *"Don't want to miss out on selection, don't want to not get paid for that training session".*<br>• *"I don't want to seem weak".*	

Copy of the Online Questionnaire

The Role of the Menstrual Cycle on Sports Performance

Introduction

The Role of the Menstrual Cycle on Sports Performance

Welcome and thank you for considering taking part in this online survey.

Before you decide, we would like you to understand why the research is being done and what it would involve for you. Please contact one of the investigators using the contact details below if you have any questions.

The purpose of this study is to gain information relating to the menstrual cycle and how it affects rugby performance. The study aims to find out to what extent, if any, the

menstrual cycle effects Sports Performance, how Elite Female Athletes are affected, and if there are any successful interventions to be put in place to prevent performance reductions.

This study is part of a student research project supported by Loughborough University. The study will be undertaken by Eloise Hayward and supervised by Sarabjit Mastana.

You will be asked to complete an anonymous online survey, which should take no longer than 10 minutes to complete. You do not need to do anything before completing the survey. This is a low-risk activity and no disadvantages or risks have been identified in association with participating.

You must be over the age of 18 and have the capacity to fully understand and consent to this research. You must also be an Elite level female rugby player, currently playing in or have played in the Allianz Premier 15s or Women's Betfred Super League.

Loughborough University will be using information/data from you to undertake this study and will act as the data controller for this study. This means that the University is responsible for looking after your information and using it properly. No identifiable personal information will be collected and so your participation in the study will be confidential. The anonymous data will be used in student dissertations. No individual will be identifiable in any report, presentation, or publication. All information will be securely stored on the University computer systems. Anonymised data will be retained until the final project marks have been verified, expected July 2023.

After you have read this information and asked any questions you may have, if you are happy to participate, please read the consent page and confirm your consent by checking the tick box at the bottom of the page. You can withdraw from the survey at any time by closing the browser. However, as the survey is anonymous once you have submitted the survey it will not be possible to withdraw your data from the study.

Sarabjit Mastana (Responsible Investigator), School of Sport, Exercise and Health Sciences, Loughborough University, Loughborough, Leicestershire, LE11 3TU, s.s.mastana@lboro.ac.uk, 01509223041

Eloise Hayward (Main investigator), School of School of Sport, Exercise and Health Sciences, Loughborough University, Loughborough, Leicestershire, LE11 3TU, e.hayward-21@student.lboro.ac.uk

What if I am not happy with how the research was conducted?

If you are not happy with how the research was conducted, please contact the Secretary of the Ethics Review Sub-Committee, Research & Innovation Office, Hazlerigg Building, Loughborough University, Epinal Way, Loughborough, LE11 3TU. Tel: 01509 222423. Email: researchpolicy@lboro.ac.uk

The University also has policies relating to Research Misconduct and Whistle Blowing which are available online at https://www.lboro.ac.uk/internal/research-ethics-integrity/research-integrity/ .

If you require any further information regarding the General Data Protection Regulations, please see: https://www.lboro.ac.uk/privacy/research-privacy/

Consent

Informed Consent Section The purpose and details of this study have been explained to me. I understand that this study is designed to further scientific knowledge and that all procedures have received a favourable decision from the Loughborough University Ethics Review Sub-Committee. I have read and understood the information sheet and this consent form. I have had an opportunity to ask questions about my participation. I understand that taking part in the survey is anonymous, only non-identifying demographic information will be collected, e.g. gender. I understand that I am under no obligation to take part in the study and can withdraw during the survey by closing the browser but will not be able to withdraw once my responses have been submitted. I understand that information I provide will be used for the student's dissertation.
Consent to Participate I voluntarily agree to take part in this study.

- ○ Yes

Question 1&2

How old are you?

- 18-24
- 25-31
- 32-38
- 38-44

Do you track your period?

- Yes
- No

If yes, how? What app or method do you use?

Question 3&4

What's your typical period length? (days in which you bleed)

What is your average cycle length? (average amount of days until you bleed again/start a new period)

Question 5&6

Have you, in the past, gone for any length of time without menstruating regularly?

- Yes
- No

If yes, for how long?

If you answered yes to the above question, what were the circumstances that were present during this time? (exercising excessively, inadequate nutrition, other stressors). Please be specific.

Question 7

What symptoms do you experience during menses? Rate the effect of your symptoms (scale of 1-5) – effects on normal life and performance/training.

	1 – no effect, 2 – some effect, 3 – noticeable effect, 4 – significant effect, 5 – dramatic effect				
	1	2	3	4	5
Abdominal cramps/pain	○	○	○	○	○
Fatigue or Low Energy	○	○	○	○	○
Bloating or Fluid Retention	○	○	○	○	○
Mood swings	○	○	○	○	○
Headaches	○	○	○	○	○
Joint pain or muscle aches	○	○	○	○	○
Constipated or Diarrhoea	○	○	○	○	○
Trouble sleeping	○	○	○	○	○
Acne	○	○	○	○	○
Tender breasts	○	○	○	○	○
Lower back pain	○	○	○	○	○
Feeling irritable, depressed or anxious	○	○	○	○	○
Decreased motivation	○	○	○	○	○

Other symptoms and their effect? Please state.

Question 8&9

Do you currently take hormonal contraceptives?

- Yes – Combined pill
- Yes – Implant
- Yes – Injection
- Yes – Coil Yes
- – Patch
- Yes – Progestogen only pill Yes –
- Other
- No

If take other form of contraceptive, please state.

Have you ever been treated for irregular periods or heavy bleeding?

- Yes
- No

If yes, please specify details. Treatment, reasons for why this happened etc.

Question 10&11

Have you ever been injured whilst on your period or about to start?

- Yes
- No

If yes, please state the injury, the time it took to return, and treatment

Have you ever considered your period being the reason for injury?

- Yes
- No

If yes, please state the injury, the time it took to return, and treatment, and why you believe your period to be a leading factor

Question 12&13

Do you consider your playing/training performance to be affected whilst on your period?

- Yes
- No

If Yes, how?

With relation to training and playing, does your period mentally/psychologically affect you?

- Yes
- No

If yes, how? (White shorts, feel slower mentally and physically, symptoms distracting, fear of flooding, motivation, emotional etc.)

Question 14&15

With relation to training and playing, does your period physically affect you?

- Yes
- No

If yes, how? (Gym, feel slower, speed, strength, endurance, power etc.)

With relation to training and playing, does your period nutritionally affect you?

- Yes
- No

If yes, how? (Eat more, eat less, feel sick, decreased appetite, eat more chocolate)

Question 16,17&18

Do you think you’d benefit from period management education around training/playing?

- Yes
- No

If yes, what would you like to know? (nutrition, lifestyle changes, gym management, prehab/preventatives, medications)

Would you like your period to be considered in training/playing loads?

- Yes
- No

If yes, what sort of things would you like to be considered?

Would you like a generic/individual plan from staff in place to manage you on your period?

- Yes
- No

If Yes, what sort of things would you like to see in the plan?

Question 19, 20 &21

How much education have you had on periods?

- None
- Not a lot
- Some
- Enough
- A lot

Where was it from?

- School
- Sport
- Work
- Self-research Other

If you selected Other, please specify:

Do you feel comfortable speaking to a member of staff regarding your period and cycle effects?

- Yes
- No

If yes, please define their role and gender (eg., female physio). Please include all

members of staff

Do you feel comfortable taking time off due to your period?

- Yes
- No

Please explain reasoning. E.g. scared it'll affect selection or I have an understanding coach

Question 22&23

Do you feel more at risk of injury on your period?

- Yes
- No

If yes, why?

Do you feel you have ever been not selected for a game/during a trial due to your period affecting training performance?

- Yes
- No

Question 24 &25

Do you consider your performance to be better, worse or same on your period?

- Better
- Same
- Worse

Please specify, why?

Does your weight change during your menstrual cycle?

- Yes – increases Yes –
- decreases No – stays
- the same

Final page

Thank you for completing this survey and your time! Your answers have been anonymously recorded and will be great help in reporting the effects of the menstrual cycle on injuries and sports performance!

Thank you! Merci! Diolch!

Key for selection options

4 - What's your typical period length? (days in which you bleed)

1
2
3
4
5
6
7
8+

5 - What is your average cycle length? (average amount of days until you bleed again/start a new period)

Below 25
25
26
27
28
29
30
31

32
33
34
35+
